# Acute hepatitis C – from the time of diagnosis up to six months of follow-up: a cohort retrospective study

**DOI:** 10.1186/1471-2334-14-S7-P5

**Published:** 2014-10-15

**Authors:** Valeriu Gheorghiță, Alina Elena Barbu, Loredana Benea, Florin Alexandru Căruntu

**Affiliations:** 1 Central Universitary Emergency Military Hospital Dr Carol Davila, Bucharest, Romania; 2 National Institute for Infectious Diseases "Prof. Dr. Matei Balş", Bucharest, Romania; 3 Carol Davila University of Medicine and Pharmacy, Bucharest, Romania

## Background

Worldwide, the hepatitis C virus (HCV) infection is a common cause of chronic liver disease, but it is rarely diagnosed in the stage of acute hepatitis. Although hepatitis C was the most important hepatitis related to blood products transfusion, since 1992 the overall epidemiology of HCV infection has changed dramatically, many newly diagnosed cases of HCV infection being related to the intravenous drugs use.

## Methods

We conducted a cohort, retrospective study on all cases of acute hepatitis C admitted in the National Institute for Infectious Diseases “Prof. Dr. Matei Balş”, Bucharest, over a period of about 5 years. We aimed to update, in our population, the epidemiological data, laboratory profile and natural evolution during six months after establishing the diagnosis of acute hepatitis C. We analyzed demographic data, risk factors for HCV infection and lab tests at diagnosis: the platelet count, prothrombin concentration (PC), transaminases, bilirubin level, titer of anti-HCV total antibodies and HCV viral load. Thereafter, we checked the serum HCV-RNA at 3 and 6 months, in order to estimate the rate of spontaneous viral clearance. Some follow-up missing data have been obtained through telephone interviewing of the patients.

## Results

Between January 2010 and July 2014, we recorded a number of 31 patients. The median age of the patients was 45 years (IQR 37, 60) and 54.8% were male. In 2013 there was an increase in the number of diagnosed cases (n=9, 29%) compared to previous year (n=3, 9% in 2012). The main route of transmission of HCV infection was related to medical and surgical exposure in the past (n=17, 54.8%). Only 2 cases (6.4%) were related to intravenous drugs use. Most patients presented clinical jaundice at diagnosis (n=19, 61.2%). The median bilirubin level was 9.5 mg/dL. Most cases were mild hepatitis (n=29, 93%), with a median PC of 96%. In only a single case the PC was 26% from the beginning. Anti-HCV total antibodies were positive in 93.5% of patients; the median serum level was 10.3 IU/mL. The average value of HCV-RNA at diagnosis was 6.8 log_10_ IU/mL. The rate of spontaneous viral clearance at six months of follow-up was 50%.

## Conclusion

Our study showed that, in cases of hospitalized acute hepatitis C, medical and surgical exposures were the major routes of HCV infection, anti-HCV serum antibodies were positive at the time of diagnosis in almost all cases and half of the patients achieved spontaneous viral clearance.

